# Possible Obesogenic Effects of Bisphenols Accumulation in the Human Brain

**DOI:** 10.1038/s41598-018-26498-y

**Published:** 2018-05-29

**Authors:** Pantelis Charisiadis, Xanthi D. Andrianou, Thomas P. van der Meer, Wilfred F. A. den Dunnen, Dick F. Swaab, Bruce H. R. Wolffenbuttel, Konstantinos C. Makris, Jana V. van Vliet-Ostaptchouk

**Affiliations:** 1 Cyprus International Institute for Environmental and Public Health, Cyprus University of Technology, Limassol, 3041 Cyprus; 2 Department of Endocrinology, University of Groningen, University Medical Center Groningen, Groningen, The Netherlands; 3 Department of Pathology & Medical Biology, University of Groningen, University Medical Center Groningen, Groningen, The Netherlands; 4 Netherlands Institute for Neuroscience, an Institute of the Royal Netherlands Academy of Arts and Sciences, Amsterdam, The Netherlands

## Abstract

Evidence of bisphenols’ obesogenic effects on humans is mixed and inconsistent. We aimed to explore the presence of bisphenol A (BPA), bisphenol F (BPF) and chlorinated BPA (ClBPA), collectively called the bisphenols, in different brain regions and their association with obesity using *post-mortem* hypothalamic and white matter brain material from twelve pairs of obese (body mass index (BMI) >30 kg/m^2^) and normal-weight individuals (BMI <25 kg/m^2^). Mean ratios of hypothalamus:white matter for BPA, BPF and ClBPA were 1.5, 0.92, 0.95, respectively, suggesting no preferential accumulation of the bisphenols in the grey matter (hypothalamic) or white matter-enriched brain areas. We observed differences in hypothalamic concentrations among the bisphenols, with highest median level detected for ClBPA (median: 2.4 ng/g), followed by BPF (2.2 ng/g) and BPA (1.2 ng/g); similar ranking was observed for the white matter samples (median for: ClBPA-2.5 ng/g, BPF-2.3 ng/g, and BPA-1.0 ng/g). Furthermore, all bisphenol concentrations, except for white-matter BPF were associated with obesity (*p* < 0.05). This is the first study reporting the presence of bisphenols in two distinct regions of the human brain. Bisphenols accumulation in the white matter-enriched brain tissue could signify that they are able to cross the blood-brain barrier.

## Introduction

During the past four decades, a dramatic increase in the global prevalence of obesity has been documented, with estimates of 641 million obese individuals in 2014, worldwide, versus 105 million in 1975^1^. It is widely accepted that the main driver of this obesity epidemic is modern lifestyle that combines excessive caloric intake with sedentary behaviour. The response to the ‘obesogenic’ environment is further modulated by individual genetic predisposition to gain weight^2^. However, the rapid pace at which the obesity epidemic occurs cannot be solely explained by these established risk factors^3^. A growing body of evidence suggests that exposure to certain environmental pollutants called endocrine-disrupting chemicals (EDC) may be another important contributor to the development of obesity^4^.

Obesity is a complex endocrine disorder characterized by disruption of multiple hormonal systems involved in the control of body metabolism^3,4^. The recent scientific declaration called Parma consensus defined the metabolic disruptors such as EDC that may interfere with hormone actions play a key role in altering susceptibility to obesity and related chronic diseases including metabolic syndrome and diabetes^3^. The most prominent example of such metabolic disruptors includes various bisphenols that are extensively used in epoxy resins and other plastic products^5^. The major bisphenols are: (i) bisphenol A (BPA), one of the highest volume production chemicals in the world, (ii) BPA structural analogs, usually substituting BPA in polymeric matrices of commercial formulations such as bisphenol F (BPF) or bisphenol S (BPS), and (iii) chlorinated BPA derivatives (ClBPA) formed when BPA in the environment reacts with disinfectant chlorine or chlorinated oxidants^6^. The widespread detection of these chemicals in human biospecimen suggests ubiquitous exposures to bisphenols^5,7^. Animal and *in-vitro* studies have highlighted the metabolic effects of bisphenols, with BPA analogs or derivatives showing equal, if not greater endocrine disruptive activity than that of BPA^6,8,9^.

Evidence of bisphenols’ obesogenic effects on humans is mixed and inconsistent. While epidemiological studies reported positive associations between BPA exposures and obesity in adults, children and adolescents^10–12^, major controversy continues about how to link the concentrations of short half-lived BPA detected in human samples with adverse health effects. To date, a few biological modes of action have been proposed by which bisphenols may exert changes in body weight, including binding to thyroid receptor^13^ and alteration of thyroid hormone levels (reviewed by Andra and Makris^14^); activation of PPARy^15^ and glucocorticoid receptors^16^; regulation of adipocyte differentiation^17^ and absorption of lipids by adipocytes^18^. BPA is also shown to have insulinotropic action and adverse effects on glucose metabolism through estrogen receptors^19,20^. Altogether, the data indicate the high potency of bisphenols to interfere with a wide range of endocrine physiological networks.

One of the potential endpoints for bisphenols action on metabolism may be the hypothalamus, a brain structure that plays a crucial role in energy balance control^21,22^. The hypothalamic infundibular nucleus is the central area of metabolic regulation, consisting mainly of grey matter^23,24^. However, before reaching the inner brain areas (e.g. white matter and lipid-enriched areas), bisphenols have to cross the blood-brain barrier that generally protects the brain from exposures to harmful substances, including toxins and bacteria^25^. Scarce data of passage across the blood-brain barrier exist for compounds, such as methylated mercury or lead ions^25^. These charged ions have been shown to cross through the endothelial cells of the blood-brain barrier under certain circumstances^26^. So far, such evidence is lacking for bisphenols in human studies.

To assess the potential accumulation of bisphenols in different regions of the human brain and their possible interference with central regulation of body weight, we examined the presence of BPA, BPS and ClBPA in hypothalamic and white matter-enriched areas and their association with obesity.

## Materials and Methods

### Study Design and Tissue Sample Collection

Brain samples were obtained from the Netherlands Brain Bank (NBB) Netherlands Institute for Neuroscience (open access: www.brainbank.nl). All material has been collected from donors for or from whom a written informed consent for a brain autopsy and the use of the material and clinical information for research purposes was obtained by the NBB.

A case-control study was set up after post-mortem hypothalamic and white matter material was obtained by autopsy from 24 individuals. Hypothalami were selected from twelve obese (cases) and twelve normal-weight (controls) individuals (defined as body mass index (BMI) >30 and BMI <25 kg/m^2^, respectively), matched for sex, age, clinical diagnosis and Braak stage of Alzheimer progression^27^; white matter-enriched brain tissue was also available and further used for twelve of these subjects.

Four extra subjects had multiple tissues freshly-obtained, such as hypothalamus, omentum central fat (visceral fat) and skin fat tissues. Samples of fat were collected from the subcutis of the abdomen as well as from the *omentum majus*. In addition, the hypothalamus was dissected from the left hemisphere. Post-mortem delay was in all 4 cases less than 24 hours; for the first case we had informed consent, because of research conducted in our center for ataxic disorders. For the rest 3 patients, we followed the National Code of Good Use of patient material, which is supported by the Medical Ethical Committee of the UMCG. The available clinic-pathological characteristics of the subjects can be found in Supplementary Table S1A,B.

All procedures were performed in accordance with national and institutional guidelines and with the ethical guidelines of the Declaration of Helsinki.

### Bisphenols Biomonitoring in Brain and Fat Tissues

The bioanalytical methodology for analyzing BPA, BPF and ClBPA in brain and adipose tissues was modified after the methods of Termopoli *et al*.^28^ and the MAK-collection for occupational health and safety^29^. Bisphenol A (BPA) was purchased from Sigma-Aldrich, USA; Bisphenol F (BPF) from AccuStandard, New Haven, CT, USA; 3-chlorobisphenol A (ClBPA) from Santa Cruz Biotechnology Inc. Santa Cruz, CA, USA; the internal standard bisphenol A-d16 (ISTD) from Supelco, USA; ethyl acetate (EtOAc) analytical grade from Fisher Chemical, UK, while hexane (Hex), methanol, acetone and dichloromethane were all GC grade from Sigma-Aldrich, Germany, and trifluoroacetic anhydride (TFAA) from Merck, Germany. The enzyme β-glucuronidase *Helix pomatia* from Calbiochem, UK, and sodium acetate trihydrate from Sigma-Aldrich, Germany. Lamb brain was purchased from a local meat shop.

Stock solutions of 1000 mg/L were prepared from the initial standard compounds in methanol, and further diluted to prepare calibration and additive solutions. A solution of 1000 mg/L of bisphenol A-d16 in methanol was prepared. All solutions were kept away from light, prepared fresh, and stored always at −20 °C in glass vials. An acetate buffer solution of pH 5.0 (0.5 M) was prepared in distilled water from sodium acetate trihydrate. The buffer solution was stored at 4 °C in glass vial. Ethyl acetate and hexane were distilled prior to use. Only glass pipettes and glass syringes were used throughout the experiments.

#### Sample preparation and extraction

Frozen brain samples were thawed at room temperature, and cut into pieces of ~0.50 ± 0.02 g each. Then, transferred in 50 mL polypropylene screw top vials with screw cap and thawed at room temperature, following the addition of 14 mL distilled water and 1 mL of buffer solution. The mixture was homogenized in a laboratory electrical mixer for 1 minute at 10000 rpm, following the addition of 5 KU of the enzyme β-glucuronidase *Helix pomatia*. The vial was shaken in vortex and incubated for 3-h at 37 °C. After, the mixture was cooled to room temperature and centrifuged for 3 minutes at 3000 rpm, and 7 mL of the upper aqueous layer was transferred in 15 mL screw top glass vials. Then, addition of 70 μL of internal standard solution of 3.0 mg/L (at total amount of 200 ng and at final brain concentration of ~500 ng/g), and extraction with 2.5 mL EtOAc:Hex (1:4) solution by vigorous shaking in a laboratory shaker for 15 min at 135 rpm. Phase separation was facilitated by centrifuge of the sample for 1 min at 2500 rpm. From the upper organic phase, 1.5 mL was collected in a GC vial and solvent was evaporated under gas nitrogen stream. All glass syringes were washed with acetone in between samples. After solvent evaporation, samples were placed in the oven at 90 °C for 15 minutes to remove any residual water. The dried samples were capped and cooled to room temperature. The sample was reconstituted with 100 μL of EtOAc:Hex (1:4) followed by the addition of 100 μL of TFAA, gently shaken in vortex and waiting for 30 min for reaction completion. After derivatization was completed, samples were taken to dryness once again, under gas nitrogen stream, in order to remove excess of TFAA. The final solution for GC-MS/MS measurements was produced by the addition of 400 μL dichloromethane. The vials were recapped and gently mixed in a vortex.

#### GC-PTV-QqQ-MS/MS analysis

GC-MS spectra were recorded on an Agilent 7890 A GC equipped with an Agilent 7000 B triple quadrupole MS detector and programmed temperature vaporization (PTV) injector. A 20 μL sample were injected at a rate of 100 μL/min while the inlet was maintained at 35 °C for 0.35 min, ramped to 300 °C at a rate of 300 °C/min. The injection syringe was washed 3 times with acetone before and after sample injection. Solvent was evaporated for 0.3 min at a flow rate 30 mL/min at 0 psi and after 1.5 min of sample loading on the column; the purge valve was opened at a flow 30 mL/min. Compounds were separated on the Rxi-5 ms (5% diphenyl/95% dimethylpolysiloxane) column from Restek (30 m × 250 × 0.25 μm). Helium carrier gas (99.999%) flow was maintained at 1.0 mL min^−1^. The oven was set to 30 °C for 1.5 min, ramped to 220 °C at a rate 40 °C min^−1^ where it was maintained for 5 min, then ramped to 300 °C at a rate 80 °C min^−1^ where it was maintained for 1.75 min followed by a post run period at 300 °C for 1 min. MSD transfer line and MS source temperatures were held at 250 °C, while quadruples were held at 150 °C. Mass spectra were obtained using electron impact ionization (70 eV), in the multiple reaction monitoring (MRM) mode, with a solvent delay of 8.0 min. The system was controlled by the software Mass Hunter Workstation (Agilent, rev. B.05.00). The parent-daughter ion pairs used for multiple reaction monitoring were chosen, based on ion abundances in different collision energies while scanning cycles were kept constant to approximately 3 cycles per second. In brief, mass spectra of a standard solution were obtained and analytes were identified by comparison to NIST mass spectra library (NIST 08 version 2.0). For each compound, molecular ion MS/MS spectra in different collision energies were obtained. Collision energies for the chosen parent-daughter ion pairs of each analyte were optimized to achieve maximum signal intensity.

#### Calibration curve and quality control

Procedural calibration standards were prepared in lamb brain samples. A portion of 0.50 ± 0.02 g from the lamb brain sample was prepared as before. The desired concentration dilution standards in methanol were added after the internal standard solution addition step. The curves were established by measuring ten samples at final concentration from 0.2 to 83.0 ng using GC-PTV-QqQ-MS/MS in the MRM mode. Quantitative analysis was based on peak area measurements as ratios versus peak area of internal standard. The limit of detection (LOD) was determined at the concentration level of 2.5 ng for each analyte. The limit of quantification (LOQ) and LOD were determined based on the standard deviation of nine measurements of the spiked samples. LOD was calculated by adding 3x the standard deviation of the lowest response concentration of the calibration curve. Accordingly, LOQ was equal to 3x the LOD. The LOD values for BPF, BPA, and ClBPA were 0.5, 0.4, and 0.7 ng/g, respectively, in the same range as in the original method^28^. In order to evaluate the current methodology, lamb brain samples were spiked with a methanol solution of standards compounds at a final concentration of 5.0, 20.0 and 40.0 ng/g. The spike recovery values were 116 ± 8%, 94 ± 7%, and 82 ± 10% for BPF, 92 ± 4%, 91 ± 4%, and 85 ± 5% for BPA, 101 ± 17%, 85 ± 3%, and 85 ± 8% for ClBPA, respectively (n = 3 replicates).

All procedures were performed in accordance with national and institutional guidelines and with the ethical guidelines of the Declaration of Helsinki.

### Statistical analysis

Data for continuous variables were presented as median (interquartile range IQR), unless stated otherwise. Differences between groups were evaluated by the nonparametric Wilcoxon test. Descriptive characteristics of the study participants (mean, standard deviation, median, mix, max and percentiles) of BPF, BPA, and ClBPA were presented. Bisphenols concentrations in tissues were reported as ng/g and the corrected bisphenols values for body weight [body weight corrected concentration (noted as ng/g BW corr) = raw ng/g of tissue/body weight in kg*100]. Spearman correlation coefficients were calculated between the raw compound levels and age, body weight, BMI and other parameters. The descriptive statistics were repeated after the exclusion of an outlier (control sample) from the hypothalamus sample concentrations. For eleven participants both hypothalamus and white matter samples were available, thus, the corresponding paired Wilcoxon tests were performed. Statistical analyses were conducted with R (version 3.3.3) in RStudio (version 1.1.136) using the packages: tableone, dplyr, data.table; the fitted curves for the correlations between BMI and bisphenols concentrations have been constructed using the LOESS (locally weighted smoothing) method from the ggplot2 R-package^30–35^. Box and whisker plots were computed with GraphPad Prism software for Windows, Version 5. Tests were two-tailed. The level of a nominal significance was set at *P* < 0.05. All *p*-values should be interpreted with caution as the exact significance could not be estimated due to ties in the rankings used in the non-parametric tests as the sample sizes were small.

## Results

### Detection of Bisphenols in hypothalamic and white matter enriched brain tissues

Hypothalamic (HYP) and white matter-enriched areas (WM) of brain tissue samples were *post mortem* obtained from twelve obese (cases) and twelve normal-weight (control) individuals (Table 1). The mean age of the study population was 74 years old, being mostly females (65%). Brain weight and storage time of tissues were not significantly different (*p* > 0.05) between cases and controls (Table 1).

**Table 1:** Basic phenotypic characteristics of the study participants. Data given as mean (SD).

	HYP:case	WM:case	HYP:control	WM:control
N	12	6	11	6
Age (yrs)	74.5 (13.8)	73.7 (15.3)	72.7 (15.5)	71 (16)
Height (cm)	162 (7)	163 (7)	168 (12)	167 (7)
Body weight (kg)	86.2 (7.9)	87 (7.7)	64 (9.3)	63.8 (7.2)
BMI (kg/m^2^)	32.8 (2.2)	32.8 (2.5)	22.4 (1.6)	22.7 (1.5)
Brain weight (g)	1130 (130)	1146 (154)	1182 (123)	1190 (132)
Storage time (yrs)	9.8 (5.7)	8.0 (5.0)	5.6 (2.0)	7.3 (4.0)

BMI: body mass index; HYP: hypothalamus; WM: white matter enriched brain area; yrs: years.

Except for one sample, all hypothalamic samples had detectable levels for all three bisphenols. External contamination for bisphenols in the lab or during shipping of samples was minimal, evident by acceptable method blanks and spike recoveries (Supplementary Tables S2 and S3). The distribution of bisphenols in hypothalamus was skewed with ClBPA [2.4 (2.3, 2.55) ng/g] showing the highest median(IQR) levels, followed by BPF [2.2 (2.1, 2.8) ng/g] and BPA [1.2 (1.0, 2.35) ng/g]; a similar trend was observed for the WM samples, where ClBPA levels were highest [2.5 (2.3, 2.52) ng/g] followed by BPF [2.3 (2.0, 2.73) ng/g] and BPA [1.0 (1.0, 1.75) ng/g] (Table 2). The levels of bisphenols in both hypothalamic and WM samples stratified by sex and status are provided in Table S4. A positive correlation between BPA and BPF in hypothalamic and white-matter brain areas was found (*r* = 0.84, *P* < *0.001*, and *r* = 0.73, *P* < 0.05) (Table 3). There was no significant (*P* > 0.05) difference in the bisphenols levels between hypothalamic and white matter-enriched brain areas, suggesting that bisphenols were able to cross the blood brain barrier, translocating beyond the grey matter structures of the human brain (Tables 2 and 4, Table S5, Figure S1). In specific, the mean ratios of hypothalamus:white matter for BPA, BPF and ClBPA were 1.5, 0.92, 0.95, respectively, suggesting no preferential accumulation of the more lipophilic BPF and ClBPA; BPA, the least lipophilic of all three bisphenols was preferentially accumulated in the hypothalamus.

**Table 2:** Descriptive statistics of the compounds (ng/g) for the whole study population and by status (case/control) for the different brain tissue types.

	Status	n	Mean	SD	Median	Min	p25	p75	Max	*P*-value
**Hypothalamus**										
BPF		23	3.95	6.06	2.2	1.7	2.1	2.80	30.8	
BPA		23	2.52	2.95	1.2	0.9	1.0	2.35	14.5	
ClBPA		23	4.17	6.41	2.4	2.2	2.3	2.55	32.8	
BPF	case	12	2.21	0.35	2.15	1.7	2.05	2.35	2.8	0.07
	control	11	5.85	8.54	2.40	1.9	2.20	4.25	30.8	
BPA	case	12	1.67	1.27	1.15	0.9	1.00	1.88	5.4	0.13
	control	11	3.45	3.94	2.20	0.9	1.15	3.75	14.5	
ClBPA	case	12	2.36	0.11	2.30	2.2	2.30	2.40	2.6	0.14
	control	11	6.15	9.06	2.50	2.2	2.30	4.75	32.8	
**WM brain**										
BPF		12	2.40	0.51	2.3	1.7	2.0	2.73	3.4	
BPA		12	1.65	1.37	1.0	0.9	1.0	1.75	5.7	
ClBPA		12	2.53	0.36	2.5	2.3	2.3	2.52	3.6	
BPF	case	6	2.37	0.29	2.30	2.0	2.20	2.63	2.7	1.00
	control	6	2.43	0.69	2.40	1.7	1.85	2.88	3.4	
BPA	case	6	1.22	0.49	1.00	0.9	1.00	1.15	2.2	0.51
	control	6	2.08	1.85	1.30	0.9	1.00	2.12	5.7	
ClBPA	case	6	2.45	0.12	2.50	2.3	2.35	2.50	2.6	0.93
	control	6	2.62	0.50	2.45	2.3	2.32	2.58	3.6	

BPA: bisphenol A; BPF: bisphenol F; ClBPA chlorinated BPA derivatives; WM: white matter-enriched brain region. *P*-values < 0.05 are indicated in bold.

**Table 3:** Spearman correlation coefficients between the compound levels, age, and BMI for the samples of the different tissue types.

	BPF (ng/g)	BPA (ng/g)	Age (yrs)	Height (cm)	Weight (kg)	BMI (kg/m^2^)	Brain weight (g)	Storage time (yrs)
**Hypothalamus (n = 23)**								
BPF (ng/g)			0.02	0.09	−0.21	−0.32	0.08	0.12
BPA (ng/g)	0.84****		0.10	0.14	−0.22	−0.29	−0.06	0.07
ClBPA (ng/g)	0.38	0.41	−0.19	0.00	−0.31	−0.31	0.31	−0.21
**WM brain (n = 12)**								
BPF (ng/g)			0.02	−0.05	−0.04	−0.22	0.28	−0.15
BPA (ng/g)	0.73**		0.00	−0.12	−0.39	−0.47	0.21	−0.08
ClBPA (ng/g)	0.33	0.17	0.07	0.48	0.15	−0.20	0.12	−0.27

****P < 0.0001; ***P < 0.001, **P < 0.01, *P < 0.05.

BPA: bisphenol A; BPF: bisphenol F; ClBPA: chlorinated BPA derivatives; WM: white matter-enriched brain area; yrs: years.

**Table 4:** Paired comparison of the hypothalamus and WM concentrations (median, IQR) for the participants with both samples. *P*-values of the paired Wilcoxon test are presented.

	Hypothalamus	WM	*P-*value
N	11	11	
BPF (ng/g)	2.20 [2.05, 2.35]	2.40 [2.10, 2.75]	0.13
BPA (ng/g)	1.20 [1.00, 1.90]	1.00 [1.00, 1.90]	1.00
ClBPA (ng/g)	2.40 [2.30, 2.45]	2.50 [2.35, 2.55]	0.28
	**Hypothalamus**	**WM/LP**	***P*** **-value**
N	11	11	
BPF (ng/g, BW corr)	2.97 [2.54, 3.35]	3.07 [2.66, 3.96]	0.24
BPA (ng/g, BW corr)	1.67 [1.40, 2.38]	1.36 [1.16, 2.59]	0.96
ClBPA (ng/g, BW corr)	2.97 [2.66, 3.58]	3.42 [2.77, 3.97]	0.34

BPA: bisphenol A; BPF: bisphenol F; ClBPA chlorinated BPA derivatives; BW corr: body weight corrected; WM: white matter-enriched brain region.

### Levels of Bisphenols and obesity

Stratifying tissue data by obesity status, median bisphenols levels (ng/g) in hypothalamus were all consistently higher in controls than in obese subjects; same trend was also observed for the WM tissues (Table 2). When calculated a concentration per body weight (in kg) to account for compound ‘dilution’ effects due to a larger body weight, the median bisphenols levels in obese individuals remained lower than their control counterparts, being all significantly (*p* < 0.05) lower in brain tissue samples of obese individuals (Table 5). A negative correlation was observed between all bisphenols concentrations in both hypothalamic and WM tissues with body weight or BMI (Table 3).

**Table 5:** Body weight corrected bisphenols levels for different brain tissues for the whole study population and by obesity status (case/control).

	Status	n	Mean	SD	Median	Min	p25	p75	Max	*P*-value
**Hypothalamus**										
BPF (ng/g, BW corr)		23	5.80	10.16	3.18	2.00	2.47	3.92	51.33	
BPA (ng/g, BW corr)		23	3.71	5.01	1.83	1.03	1.36	3.52	24.17	
ClBPA (ng/g, BW corr)		23	6.14	10.78	3.14	2.37	2.71	4.36	54.67	
BPF (ng/g, BW corr)	case	12	2.58	0.45	2.47	2.00	2.25	2.95	3.29	**0.0003**
	control	11	9.31	14.17	4.00	2.97	3.40	7.07	51.33	
BPA (ng/g, BW corr)	case	12	1.93	1.42	1.40	1.03	1.16	2.14	6.14	**0.011**
	control	11	5.66	6.70	3.38	1.49	1.75	5.95	24.17	
ClBPA (ng/g, BW corr)	case	12	2.76	0.30	2.71	2.37	2.57	2.86	3.42	**0.0001**
	control	11	9.84	15.06	4.55	2.97	3.58	7.82	54.67	
**WM brain**										
BPF (ng/g, BW corr)		12	3.30	1.02	2.94	2.25	2.56	3.65	5.37	
BPA (ng/g, BW corr)		12	2.48	2.69	1.41	1.01	1.17	2.38	10.56	
ClBPA (ng/g, BW corr)		12	3.47	0.79	3.38	2.58	2.81	3.95	4.86	
BPF (ng/g, BW corr)	case	6	2.74	0.40	2.70	2.25	2.46	3.00	3.29	0.09
	control	6	3.86	1.18	3.96	2.46	2.88	4.65	5.37	
BPA (ng/g, BW corr)	case	6	1.45	0.77	1.16	1.01	1.07	1.28	3.01	**0.026**
	control	6	3.51	3.57	1.91	1.36	1.50	3.42	10.56	
ClBPA (ng/g, BW corr)	case	6	2.84	0.31	2.77	2.58	2.63	2.85	3.42	**0.004**
	control	6	4.10	0.56	3.97	3.33	3.86	4.47	4.86	

BPA: bisphenol A; BPF: bisphenol F; ClBPA chlorinated BPA derivatives; BWcorr: body weight corrected; WM: white matter-enriched brain region. *P*-values < 0.05 indicated in bold.

The analyses were repeated without an outlier found in hypothalamus dataset (participant without a paired WM sample), but the above-observed trends remained unchanged (Table S5).

### Bisphenols in Fresh Brain and Adipose Tissues

Within freshly-obtained brain and adipose fat samples from four (4) subjects, bisphenols accumulation in skin and visceral fat tissues was compared against their corresponding accumulation in matched hypothalamic samples (Table 6). Results showed nearly double median BPF and ClBPA levels for all three compounds in hypothalamic tissue when compared with those of skin or visceral adipose tissues; however, this trend was less obvious for BPA (Table 6, Figure S2).

**Table 6:** Descriptive statistics of bisphenols concentrations (ng/g) for paired freshly-obtained hypothalamus and skin/visceral fat.

	Tissue type	n	Mean	SD	Median	Min	p25	p75	Max
BPF	Hypothalamus	3^#^	5.57	1.94	5.10	3.9	4.50	6.40	7.7
	Visceral fat	4	2.98	1.05	2.65	2.1	2.48	3.15	4.5
	Skin fat	4	2.70	0.75	2.60	1.9	2.35	2.95	3.7
BPA	Hypothalamus	3	2.67	0.49	2.90	2.1	2.50	2.95	3.0
	Visceral fat	4	1.50	0.68	1.25	1.0	1.15	1.60	2.5
	Skin fat	4	2.92	1.88	2.75	0.9	1.80	3.88	5.3
ClBPA	Hypothalamus	3	6.07	1.80	6.90	4.0	5.45	7.10	7.3
	Visceral fat	4	3.42	1.44	3.10	2.2	2.35	4.17	5.3
	Skin fat	4	2.85	0.69	2.65	2.3	2.38	3.12	3.8

^#^The hypothalamic sample for one individual was missing.

BPA: bisphenol A; BPF: bisphenol F; ClBPA: chlorinated BPA derivatives.

## Discussion

This is the first descriptive study reporting the widespread presence of BPA, BPF and chlorinated BPA compounds in both the grey matter (hypothalamic) and white matter-enriched regions of the human brain. The detection of bisphenols in the white matter-enriched brain areas signifies that these metabolic disruptors were able to cross the blood brain barrier. The mean ratio of hypothalamus:white matter was 50% higher for BPA, but not for BPF or ClBPA, suggesting that only the least lipophilic compound (BPA) was preferentially accumulated in the hypothalamus. Bisphenols tissue levels in both grey and white matter of brain areas were consistently lower in obese individuals than in those of matched controls. This observation may indicate potential interference of bisphenols with hypothalamic-centered metabolic processes related to energy balance regulation. Higher body weight could lead to lower bisphenols intake estimates per unit of body mass, because of a possible dilution effect due to larger body weight, but this was not the case even after correcting brain bisphenols data for body weight. On the contrary, the inverse association between bisphenols tissue levels and obesity (BMI) became more pronounced.

The observed negative association between bisphenol concentrations in brain tissues and obesity can be explained by potential estrogenic effect of these chemicals on body weight^36^. The role of estrogens in body weight regulation is complex and not yet fully understood, often demonstrating non-monotonic dose response curves, where similar estrogenic compounds act via a suite of sex and dose dependent mechanisms to reduce body weight and adiposity^37,38^. In addition, multiple *in vitro* studies indicate that low doses of bisphenols affect adipocyte differentiation, leading to the alterations of adipose cell function^39–41^. BPA was also shown to interfere with the development and function of the hypothalamic systems related to body weight and food intake regulation^42^. Another speculative suggestion for the lower bisphenols levels in obese samples could be the activation of enzyme systems that enhance BPA metabolism to secondary metabolites of unknown so far structure that cannot be so far detected with existing biomonitoring protocols.

All available studies of bisphenols effects on fetal, neonatal and adult brain tissues are mainly animal or *in-vitro* studies; the relevant human studies are scarce. Even at low doses, BPA was shown to disrupt neurogenesis within the hypothalamus^43^ and modified neuroendocrine axis functional targets both at central (hypothalamus and/or pituitary) and peripheral levels (thyroid, adrenal-stress and reproductive axes)^42^. Feeding behavior and metabolism are regulated by distinct neuronal circuits in the hypothalamus that produce neurotransmitters and neuropeptides, regulating food intake in response to changing energy requirements^21^. Hypothalamus is composed of mainly grey matter structures like the infundibular nucleus that are open to the blood-brain barrier. These hypothalamic structures remain crucially involved in the regulation of feeding and metabolism and apparently more “open” to circulating EDCs, like the bisphenols^25^.

To date, no human data for brain tissue BPF or ClBPA exist, except for a few studies that determined BPA in human brain and adipose tissue samples. In our study, comparable levels of BPA were detected in stored and freshly-collected hypothalamic samples (means of 2.52 ng/g (n = 23) versus 2.67 ng/g (n = 3), respectively), while BPA tissue levels in the white matter-enriched brain areas was lower (1.65 ng/g (n = 12)). We also observed similar levels of BPA in the stored hypothalamus and white-matter measured by a different analytical methodology, isotope dilution TurboFlow-LC-MS/MS^44^. Geens *et al*. reported mean BPA levels of 0.91 ng/g as measured in 8 out of 11 human brain samples^45^. Also, mean BPA in adipose tissue samples of our study (skin fat 2.92 ng/g, visceral fat 1.50 ng/g (n = 4)) were in range with those previously reported for adipose fat from two Spanish studies (0.60 ng/g, n = 6 out of 14; 5.83 ng/g, n = 11 out of 20)), Belgium (3.78 ng/g, n = 11), USA (geometric mean (GM) 3.95 ng/g, n = 18 out of 20)^45–48^. So far, only one study reported the ClBPA levels in adipose tissue with mean concentration of 3.05 ng/g detected in 3 out of 20 samples^47^, being comparable to that found in our study (2.85 ng/g in skin fat, 3.42 ng/g in visceral fat (n = 4)).

Such differences in tissue BPA concentrations between ours and those from earlier studies could reflect inter-subject variability in BPA exposure due to differences in age, sex, or ethnicity. It is plausible that the extent of bisphenols accumulation in human tissues may be dependent upon the brain area and adipose tissue type and its localisation patterns in the body. In the current study, we obtained tissues from distinct brain regions and adipose depots, all collected with the same way from all subjects. There were no details on the obtained brain and adipose tissue sampling in other human studies that reported tissue levels of xenobiotics/EDCs. To date, no BPF or chlorinated forms of BPA have been previously reported in the human brain, and in particular for the white matter-enriched brain area, so our study is the first one to demonstrate the presence of these newer versions of bisphenols in brain tissues.

It was noteworthy that obese individuals had lower bisphenols levels in brain samples than their normal-weight counterparts. This trend, earlier ascribed to reverse causality effects, has been already observed between weight and BPA concentration in the brain (brain tissue area not reported, *r* = −0.802; *P = 0.003*)^45^, as well as in urinary BPA biomarker data from children and adults in human prospective cohort studies^49–52^. Exposure to low doses of BPA during critical windows of development has been reported to have obesogenic effects in both experimental and epidemiological studies^53^. Mothers’ prenatal urinary BPA concentrations were associated with decreased BMI *z*-score, body fat, waist circumference, and obesity in their daughters; this was unexpected^50^. Similarly, early-life exposure to BPA was associated with decreased BMI at 2–5 years of age in 297 mother–child pairs^49^. Further, while no significant difference in urinary BPA concentrations was observed between overweight or obese children and those with normal weight (*P* = 0.26), BPA daily intake was unexpectedly higher among normal-weight children (*P* = 0.003)^51^. However, it must be noted that the above-mentioned studies reflect BPA-related obesogenic effects at different windows of exposure, than those in our study, i.e. the prenatal and early-life periods. Therefore, the reverse trend between BPA levels in the adult brain and obesity status observed in our study warrants further investigation. Several studies in adult animals demonstrated that low doses of BPA reduced overall energy metabolism and led to alterations in insulin action in peripheral tissues^54^ as well as influenced de novo lipogenesis in liver^55^.

Certain animal studies also showed the BMI inverse association with BPA dosing, suggesting that an alternative mechanism of (faster) BPA metabolism could be activated in obese phenotypes, where despite the high initial dosage, the resulting total urinary BPA levels decreased with obesity. Although decreased body weight in rat offspring has been reported at very high doses (>4,000 μg/kg/day)^56^, such trend has also been seeing in animal studies using environmentally-relevant doses of BPA^57–60^. Yalcin *et al*. showed that obese mice had lower activity of BPA sulfonation or glucuronidation than their wild type counterparts^61^, suggesting that Phase II metabolism could be altered by obesity. Also, the non-monotonic dose effects of BPA on adiposity and chronic inflammation, related to caloric intake have been observed in mice: BPA increased body weight and fat mass as well as systemic and adipose inflammation in animals fed a low-, but not a high-calorie diet^62^; lower BPA levels in obese animals were associated with high leptin levels showing an inverse association, where inflammation was present even in very low BPA exposures, exhibiting characteristic modes of a non-monotonic dose response curve (U-shape), where inflammation is thought of as the critical link between obesity and metabolic disease^62^.

The BPF and ClBPA accumulation in brain tissues suggests the ubiquitous exposure to such compounds. Human biomonitoring studies exist for BPF showing urinary geometric mean (GM) BPF levels of 548 ng/L (775 ng/g creatinine) and 523 ng/L (637 ng/g) in Cyprus and Romania, respectively^63^. BPF has recently attracted attention given its widespread use as a BPA substitute and its safety has been questioned^8,9^. Information on the determinants of exposure to BPF is lacking in environmental epidemiological studies. The BPA structural analogs (e.g. BPF and BPS) that considered to be safer than BPA, have recently introduced to the market and are widely used in various consumer products labelled as BPA-free. However, their long-term and adverse health effects in humans and different subpopulation groups have not been fully assessed yet. Moreover, BPA could react with disinfectant chlorine - used as disinfectant in tap water and/or consumer products- forming instantaneously chlorinated BPA derivatives (ClBPA) in the external environment that showed increased estrogen-activity (linked with some metabolic diseases) when compared with that of BPA^6,64^.

In adults, BPA is generally thought of being rapidly metabolized, being virtually complete within 24-h of exposure^65^. However, extensive non-food BPA source contribution to urinary BPA levels even when considerable fasting time elapsed since last meal, hinting to other non-ingestion-based sources of BPA exposure was documented^66^. This could hint towards longer presence and persistence of bisphenols dose in systemic circulation, allowing for further contact with brain tissues.

This study had several limitations. One of them was the relatively small sample size due to limited availability of high quality brain material from obese individuals and well-matched controls. Another limitation stemmed from the medical history of selected subjects where most of them suffered from neurological disorders that may or may not influenced the magnitude and variability of bisphenols exposure and possibly the directionality of bisphenols effects on obesity status. Since age and neurodegenerative disorders can alter the blood-brain barrier properties and permeability^67^, a potential confounding effect of these factors on the bisphenol levels observed in the examined brain regions cannot be excluded. Follow-up studies are warranted to further clarify this issue. The lack of incorporating relevant confounders in the analysis, such as dietary habits, smoking etc. was another limitation. We also cannot exclude the possibility that the measured concentrations of bisphenols were partially affected by external contamination during sample collection and storage. However, we have tested the storage containers and vials for potential contamination by bisphenols and found acceptable method blanks and spike recovery estimates. Next, there was no correlation between time of sample storage and measured bisphenols levels as well no difference in concentration between stored and freshly-obtained hypothalamic samples collected in different hospitals.

Altogether, these data suggest that bioaccumulation of bisphenols in brain tissues even those perpetually located behind the blood brain barrier, such as the white matter-enriched areas is a true finding, offering new venues for exploring hypotheses that charge such EDC with metabolic effects.

## Conclusions

Here we described for the first time the widespread presence of three bisphenols in areas of the human brain, such as the white matter-enriched area and the hypothalamus. The fact that all three bisphenols were found in hypothalamic samples suggests possible association with key processes manifested at the hypothalamus, the central regulation point of energy metabolism. Along with BPA, BPF and chlorinated BPA were detected in hypothalamic and white matter-enriched regions of the brain. These bisphenols were also shown to accumulate in skin and visceral fat of paired adipose tissue samples. Interestingly, BPF and ClBPA, but not BPA were found in lower concentrations of hypothalamus of obese individuals than those of age-, and sex-matched normal-weight subjects. The association between accumulated bisphenols in brain tissues and obesity still remains poorly understood.

This novel dataset provides hints and clues towards the design of studies that account for the possible influence of metabolic disruptors like the bisphenols on energy balance and homeostasis of the main neuroendocrine axes, both centrally and peripherally in the body. The fact that bisphenols were detected in the white matter structures of the brain, suggest crossing of the blood-brain barrier by these bisphenols having serious implications for the development of obesity and metabolic diseases. New studies are warranted to further elucidate the role of bisphenols in the development of obesity, type 2 diabetes and metabolic complications via manipulation of key hormonal centers either centrally or peripherally, especially during sensitive life stages, such as the perinatal period.

## Electronic supplementary material

Supplementary file

## References

[1] Trends in adult body-mass index in 200 countries from 1975 to 2014: a pooled analysis of 1698 population-based measurement studies with 19·2 million participants. *Lancet* **387**, 1377–1396 (2016). 10.1016/S0140-6736(16)30054-X PMC7615134 27115820

[2] van Vliet-Ostaptchouk JV, Snieder H, Lagou V (2012). Gene-Lifestyle Interactions in Obesity. Curr. Nutr. Rep..

[3] Heindel JJ (2015). Parma consensus statement on metabolic disruptors. Environ. Heal. A Glob. Access Sci. Source.

[4] Baillie-Hamilton PF (2002). Chemical toxins: a hypothesis to explain the global obesity epidemic. J. Altern. Complement. Med..

[5] Vandenberg LN, Maffini MV, Sonnenschein C, Rubin BS, Soto AM (2009). Bisphenol-A and the Great Divide: A Review of Controversies in the Field of Endocrine Disruption. Endocr. Rev..

[6] Andra SS, Charisiadis P, Arora M, Vliet-Ostaptchouk JV, Makris KC (2015). Biomonitoring of human exposures to chlorinated derivatives and structural analogs of bisphenol {A}. Environ. Int..

[7] Covaci A (2015). Urinary BPA measurements in children and mothers from six European member states: Overall results and determinants of exposure. Environ. Res..

[8] Rochester, J. R. & Bolden, A. L. Bisphenol S and F: A Systematic Review and Comparison of the Hormonal Activity of Bisphenol A Substitutes. *Environ. Health Perspect*. 10.1289/ehp.1408989 (2015). 10.1289/ehp.1408989 PMC4492270 25775505

[9] Rosenmai AK (2014). Are Structural Analogues to Bisphenol A Safe Alternatives?. Toxicol. Sci..

[10] Brent RL (2013). Bisphenol A and obesity in children and adolescents. JAMA.

[11] Carwile JL, Michels KB (2011). Urinary bisphenol A and obesity: NHANES 2003-2006. Environ. Res..

[12] Trasande L, Attina TM, Blustein J (2012). Association between urinary bisphenol {A} concentration and obesity prevalence in children and adolescents. JAMA.

[13] Jung KK (2007). Differential regulation of thyroid hormone receptor-mediated function by endocrine disruptors. Arch. Pharm. Res..

[14] Andra SS, Makris KC (2012). Thyroid disrupting chemicals in plastic additives and thyroid health. J. Environ. Sci. Health. C. Environ. Carcinog. Ecotoxicol. Rev..

[15] Phrakonkham P (2008). Dietary xenoestrogens differentially impair 3T3-{L}1 preadipocyte differentiation and persistently affect leptin synthesis. J. Steroid Biochem. Mol. Biol..

[16] Sargis RM, Johnson DN, Choudhury RA, Brady MJ (2010). Environmental endocrine disruptors promote adipogenesis in the 3T3-{L}1 cell line through glucocorticoid receptor activation. Obesity (Silver Spring)..

[17] Boucher JG, Boudreau A, Atlas E (2014). Bisphenol {A} induces differentiation of human preadipocytes in the absence of glucocorticoid and is inhibited by an estrogen-receptor antagonist. Nutr. Diabetes.

[18] Masuno H, Iwanami J, Kidani T, Sakayama K, Honda K (2005). Bisphenol a accelerates terminal differentiation of 3T3-{L}1 cells into adipocytes through the phosphatidylinositol 3-kinase pathway. Toxicol. Sci. An Off. J. Soc. Toxicol..

[19] Soriano, S. *et al*. Rapid insulinotropic action of low doses of Bisphenol-A on mouse and human islets of Langerhans: Role of estrogen receptor β. *PLoS One* **7** (2012). 10.1371/journal.pone.0031109 PMC3275611 22347437

[20] Alonso-Magdalena, P. *et al*. Pancreatic insulin content regulation by the Estrogen receptor ERα. *PLoS One* **3** (2008). 10.1371/journal.pone.0002069 PMC2323613 18446233

[21] Parker JA, Bloom SR (2012). Hypothalamic neuropeptides and the regulation of appetite. Neuropharmacology.

[22] Swaab, D. In *The Human Hypothalamus: Basic and Clinical Aspects* **79**, 476 (Elsevier, 2003).

[23] Tang Y, Purkayastha S, Cai D (2015). Hypothalamic microinflammation: a common basis of metabolic syndrome and aging. Trends Neurosci..

[24] Swaab, D. F. In *Handbook of Clinical Neurology* **80** (Elsevier, 2004).

[25] Ballabh P, Braun A, Nedergaard M (2004). The blood-brain barrier: an overview: structure, regulation, and clinical implications. Neurobiol. Dis..

[26] Fischer C, Fredriksson A, Eriksson P (2008). Neonatal co-exposure to low doses of an ortho-{PCB} ({PCB} 153) and methyl mercury exacerbate defective developmental neurobehavior in mice. Toxicology.

[27] Braak H, Braak E (1991). Neuropathological stageing of Alzheimer-related changes. Acta Neuropathol..

[28] Termopoli V, Famiglini G, Palma P, Magrini L, Cappiello A (2015). Occurrence of specific environmental risk factors in brain tissues of sudden infant death and sudden intrauterine unexpected death victims assessed with gas chromatography-tandem mass spectrometry. Anal. Bioanal. Chem..

[29] The MAK-Collection for Occupational Health and Safety, Part IV: Biomonitoring Methods. in (eds. Angerer, J. & Hartwig, A.) **12**, 169–184 (Wiley-VCH Verlag GmbH & Co. KGaA, 2010).

[30] Dowle, M., Srinivasan, A., Short, T. & Lianoglou, S. *data.table: Extension of Data.fram*e. (2015).

[31] R. *Studio: Integrated Development Environment for R*. (2015).

[32] *R: A Language and Environment for Statistical Computing*. (R Foundation for Statistical Computing, 2017).

[33] Wickham, H. & Francois, R. dplyr: A Grammar of Data Manipulation. (2015).

[34] Yoshida, K. & Bohn., J. tableone: Create ‘Table 1’ to Describe Baseline Characteristics. (2015).

[35] Wickham, H. *ggplot2: elegant graphics for data analysis*. (Springer New York, 2009).

[36] Rubin BS, Soto AM (2009). Bisphenol A: Perinatal exposure and body weight. Mol. Cell. Endocrinol..

[37] Vandenberg LN (2006). The mammary gland response to estradiol: monotonic at the cellular level, non-monotonic at the tissue-level of organization?. J. Steroid Biochem. Mol. Biol..

[38] Brown LM, Clegg DJ (2010). Central effects of estradiol in the regulation of food intake, body weight, and adiposity. J. Steroid Biochem. Mol. Biol..

[39] Verbanck M (2017). Low-dose exposure to bisphenols A, F and S of human primary adipocyte impacts coding and non-coding RNA profiles. PLoS One.

[40] Ariemma F (2016). Low-Dose Bisphenol-A Impairs Adipogenesis and Generates Dysfunctional 3T3-L1 Adipocytes. PLoS One.

[41] Wada K (2007). Life style-related diseases of the digestive system: endocrine disruptors stimulate lipid accumulation in target cells related to metabolic syndrome. J. Pharmacol. Sci..

[42] Negri-Cesi, P. Bisphenol A Interaction With Brain Development and Functions. *Dose-Response A Publ. Int. Hormesis Soc*. **13**, 1559325815590394 (2015). 10.1177/1559325815590394 PMC4674177 26672480

[43] Kinch, C. D., Ibhazehiebo, K., Jeong, J.-H., Habibi, H. R. & Kurrasch, D. M. Low-dose exposure to bisphenol A and replacement bisphenol S induces precocious hypothalamic neurogenesis in embryonic zebrafish. *Proc. Natl. Acad. Sci*. 201417731, 10.1073/pnas.1417731112 (2015). 10.1073/pnas.1417731112 PMC4321238 25583509

[44] Van Der Meer, T. P. *et al*. Distribution of non-persistent endocrine disruptors in two different regions of the human brain. *Int. J. Environ. Res. Public Health* **14** (2017). 10.3390/ijerph14091059 PMC5615596 28902174

[45] Geens T, Neels H, Covaci A (2012). Distribution of bisphenol-{A}, triclosan and n-nonylphenol in human adipose tissue, liver and brain. Chemosphere.

[46] Artacho-Cordón F (2017). Assumed non-persistent environmental chemicals in human adipose tissue; matrix stability and correlation with levels measured in urine and serum. Environ. Res..

[47] Fernandez MF (2007). Bisphenol-{A} and chlorinated derivatives in adipose tissue of women. Reprod. Toxicol..

[48] Wang L, Asimakopoulos AG, Kannan K (2015). Accumulation of 19 environmental phenolic and xenobiotic heterocyclic aromatic compounds in human adipose tissue. Environ. Int..

[49] Braun JM (2014). Early-life bisphenol a exposure and child body mass index: a prospective cohort study. Environ. Health Perspect..

[50] Harley KG (2013). Prenatal and postnatal bisphenol A exposure and body mass index in childhood in the CHAMACOS cohort. Environ. Health Perspect..

[51] Wang B (2014). Exposure to bisphenol A among school children in eastern China: a multicenter cross-sectional study. J. Expo. Sci. Environ. Epidemiol..

[52] Oppeneer SJ, Robien K (2015). Bisphenol {A} exposure and associations with obesity among adults: a critical review. Public Health Nutr..

[53] Alonso-Magdalena, P., Quesada, I. & Nadal, Á. Prenatal exposure to BPA and offspring outcomes: The diabesogenic behavior of BPA. *Dose-Response* **13** (2015). 10.1177/1559325815590395 PMC4674176 26676280

[54] Batista, T. M. *et al*. Short-term treatment with Bisphenol-A leads to metabolic abnormalities in adult male mice. *PLoS One* **7** (2012). 10.1371/journal.pone.0033814 PMC3314682 22470480

[55] Marmugi A (2012). Low doses of bisphenol a induce gene expression related to lipid synthesis and trigger triglyceride accumulation in adult mouse liver. Hepatology.

[56] Negishi T (2003). Effects of perinatal exposure to bisphenol A on the behavior of offspring in F344 rats. Environ. Toxicol. Pharmacol..

[57] Alonso-Magdalena P (2010). Bisphenol {A} exposure during pregnancy disrupts glucose homeostasis in mothers and adult male offspring. Environ. Health Perspect..

[58] Honma S (2002). Low dose effect of in utero exposure to bisphenol {A} and diethylstilbestrol on female mouse reproduction. Reprod. Toxicol..

[59] Nagel SC (1997). Relative binding affinity-serum modified access (RBA-SMA) assay predicts the relative *in vivo* bioactivity of the xenoestrogens bisphenol A and octylphenol. Environ. Health Perspect..

[60] Nakamura K (2012). Prenatal and lactational exposure to low-doses of bisphenol {A} alters adult mice behavior. Brain Dev..

[61] Yalcin EB, Kulkarni SR, Slitt AL, King R (2016). Bisphenol A sulfonation is impaired in metabolic and liver disease. Toxicol. Appl. Pharmacol..

[62] Yang M (2016). Bisphenol A Promotes Adiposity and Inflammation in a Nonmonotonic Dose-response Way in 5-week-old Male and Female C57BL/6J Mice Fed a Low-calorie Diet. Endocrinology.

[63] Andrianou XD (2016). Human Exposures to Bisphenol A, Bisphenol F and Chlorinated Bisphenol A Derivatives and Thyroid Function. PLoS One.

[64] Babu S (2012). Molecular docking of bisphenol A and its nitrated and chlorinated metabolites onto human estrogen-related receptor-gamma. Biochem. Biophys. Res. Commun..

[65] Völkel W, Colnot T, Csanády GA, Filser JG, Dekant W (2002). Metabolism and kinetics of bisphenol a in humans at low doses following oral administration. Chem. Res. Toxicol..

[66] Stahlhut RW, Welshons WV, Swan SH (2009). Bisphenol A data in NHANES suggest longer than expected half-life, substantial nonfood exposure, or both. Environ. Health Perspect..

[67] Marques F, Sousa JC, Sousa N, Palha JA (2013). Blood-brain-barriers in aging and in Alzheimer’s disease. Mol. Neurodegener..

