# Supplementary material for: Possible Obesogenic Effects of Bisphenols Accumulation in the Human Brain
Source: Sci Rep. 2018 May 29;8:8186. doi: 10.1038/s41598-018-26498-y (PMC5974368; doi:10.1038/s41598-018-26498-y)
Supplement: Supplementary file 1 — Supplementary file [file 41598_2018_26498_MOESM1_ESM.doc]

**SUPPLEMENTARY INFORMATION**

***Possible Obesogenic Effects of Bisphenols Accumulation in the Human Brain***

P Charisiadis, XD Andrianou, TP van der Meer, WFA den Dunnen, DF Swaab, BHR Wolffenbuttel, KC Makris, JV van Vliet-Ostaptchouk

**Table S1A.** Clinicopathological details of the subjects

| **NBB number** | **Age (years)** | **Sex** | **Post mortem delay (hr:min)** | **Braak stage*** | **Clinical diagnosis & Cause of death** |
| --- | --- | --- | --- | --- | --- |
| Obese group | | | | | |
| 2007-077 | 49 | M | 04:55 | 3 | Fronto-temporal dementia tauopathy |
| 2000-138 | 84 | F | 05:15 | 5 | Alzheimer's disease |
| 1998-180 | 81 | F | 04:00 | 5 | Alzheimer's disease |
| 2012-005 | 84 | F | 05:36 | 2 | Non-demented control |
| 1999-028 | 73 | M | 05:30 | 4 | Alzheimer's disease |
| 2011-066 | 81 | F | 05:55 | 4 | Parkinson's disease |
| 2013-019 | 53 | F | 07:15 | NA | Multiple sclerosis |
| 2009-071 | 77 | F | 04:30 | 0 | Fronto-temporal dementia |
| 2002-067 | 84 | F | 05:10 | 1 | Dementia |
| 2002-102 | 88 | F | 03:15 | 5 | Alzheimer's disease, type 2 diabetes |
| 2013-015 | 56 | M | 09:35 | 0 | Multiple sclerosis |
| 2009-091 | 84 | M | 07:20 | 1 | Non-demented control with Lewy bodies, type 2 diabetes |
| Control group | | | | | |
| 2013-065 | 47 | M | 05:25 | 0 | Fronto-temporal dementia |
| 2012-021 | 84 | F | 06:33 | 5 | Alzheimer's disease |
| 2009-105 | 82 | F | 05:25 | 5 | Alzheimer's disease |
| 2010-072 | 83 | F | 04:05 | 1 | Control with ischemic changes |
| 2010-120 | 71 | M | 04:00 | 6 | Alzheimer's disease with congophilic angiopathy |
| 2011-113 | 81 | F | 03:55 | 2 | Parkinson's disease |
| 2007-069 | 47 | F | 04:25 | 1 | Multiple sclerosis |
| 2002-078 | 75 | F | 04:50 | 1 | Non-Alzheimer dementia |
| 2012-058 | 84 | F | 06:45 | 2 | Vascular dementia |
| 2013-075 | 86 | F | 05:15 | 6 | Alzheimer's disease, type 2 diabetes |
| 2008-021 | 55 | M | 06:20 | NA | Multiple sclerosis |
| 2010-056 | 79 | M | 05:00 | 3 | Lewy bodies variant, type 2 diabetes |

BMI: body mass index, F: female; hr: hour; M: male; min: minutes; NA: not available; NBB: Netherlands Brain Bank; PMD: post mortem delay. * for Braak stage see the reference Braak et al., 1991 in the main text.

**Table S1B.** The data from the four subjects with freshly-obtained tissues.

| **Autopsy number** | **Age (years)** | **Sex** | **Body length (cm)** | **Abdomen circumference (cm)** | **Hip circumference (cm)** | **Cause of death** |
| --- | --- | --- | --- | --- | --- | --- |
| S16-10027 | 73 | M | 1.75 | 69 | NA | Hereditary ataxia |
| S16-10045 | 74 | M | 1.80 | 114 | NA | Lung embolism |
| S16-10039 | 65 | F | 1.60 | 89 | NA | Muscular dystrophy |
| S16-10048 | 71 | F | 1.50 | 123 | 111 | Necrotic bowel |

F: female; M: male; NA: not available

**Table S2.** Names, segments, retention time, dwell time, precursors and products ions of quantitative and confirmation transition, and collision energy for the targeted analytes

| **Analyte** | **Time** | **Retention** | **Dwell** | **Quantitative** | **Collision** | **Confirmation** | **Collision** | **Confirmation** | **Collision** | **Confirmation** | **Collision** |
| --- | --- | --- | --- | --- | --- | --- | --- | --- | --- | --- | --- |
|  | **Segment** | **Time** | **Time** | **(Q)** | **Energy** | **(q)** | **Energy** | **(q)** | **Energy** | **(q)** | **Energy** |
|  | **(min)** | **(min)** | **(ms)** |  | **(eV)** |  | **(eV)** |  | **(eV)** |  | **(eV)** |
| **BPF** | 8.000 | 8.566 | 37.5 | 279→165 | 13 | 279→153 | 41 | 392→279 | 13 | 392→165 | 35 |
| **ISTD** | 8.950 | 9.066 | 37.5 | 416→186 | 25 | 434→221 | 38 | 416→221 | 20 | 434→416 | 10 |
| **BPA** |  | 9.134 | 37.5 | 405→215 | 20 | 420→215 | 35 | 420→405 | 5 | 405→176 | 40 |
| **Cl-BPA** | 9.650 | 10.335 | 37.5 | 439→215 | 23 | 439→165 | 52 | 454→439 | 10 | 454→215 | 38 |

**Table S3.** Regression equations, linearity of calibration curves, LODs, LOQs, for the determination of the targeted analytes

|  | **Regression Equation** | **Linear Range** | **R^2^** | **LOD** | **LOQ** |
| --- | --- | --- | --- | --- | --- |
|  |  | **(ng)** |  | **(ng)** | **(ng)** |
| **BPF** | y = 0.004126 x - 0.002890 | 0.2 – 83.0 | 0.9977 | 0.2 | 0.7 |
| **BPA** | y = 0.006747 x + 0.000141 | 0.2 – 83.0 | 0.9995 | 0.2 | 0.6 |
| **Cl-BPA** | y = 0.000061 x - 0.000068 | 0.2 – 83.0 | 0.9971 | 0.4 | 1.1 |

**Table S4.** Study population descriptives and raw compound concentrations (ng/g) for the brain tissue samples by status (case or controls) and sex.

| **Hypothalamic samples**  **Compound** | **Status** | **Sex** | **n** | **Mean** | **Std Dev** | **Median** | **Min** | **p25** | **p75** | **Max** |
| --- | --- | --- | --- | --- | --- | --- | --- | --- | --- | --- |
| **BPF** | case | f | 8 | 2.21 | 0.32 | 2.15 | 1.8 | 2.05 | 2.35 | 2.8 |
|  | case | m | 4 | 2.20 | 0.45 | 2.15 | 1.7 | 2.00 | 2.35 | 2.8 |
|  | control | f | 7 | 6.67 | 10.67 | 2.40 | 2.0 | 2.25 | 3.50 | 30.8 |
|  | control | m | 4 | 4.40 | 3.37 | 3.25 | 1.9 | 2.12 | 5.52 | 9.2 |
| **BPA** | case | f | 8 | 1.43 | 0.55 | 1.15 | 0.9 | 1.00 | 1.88 | 2.3 |
|  | case | m | 4 | 2.15 | 2.17 | 1.10 | 1.0 | 1.00 | 2.25 | 5.4 |
|  | control | f | 7 | 4.27 | 4.79 | 2.20 | 1.0 | 1.40 | 4.70 | 14.5 |
|  | control | m | 4 | 2.02 | 1.23 | 1.80 | 0.9 | 1.12 | 2.70 | 3.6 |
| **ClBPA** | case | f | 8 | 2.38 | 0.13 | 2.35 | 2.2 | 2.30 | 2.42 | 2.6 |
|  | case | m | 4 | 2.32 | 0.05 | 2.30 | 2.3 | 2.30 | 2.32 | 2.4 |
|  | control | f | 7 | 7.00 | 11.40 | 2.50 | 2.2 | 2.30 | 3.45 | 32.8 |
|  | control | m | 4 | 4.67 | 2.97 | 3.90 | 2.2 | 2.58 | 6.00 | 8.7 |

| **White matter samples**  **Compound** | **Status** | **Sex** | **n** | **Mean** | **Std Dev** | **Median** | **Min** | **p25** | **p75** | **Max** |
| --- | --- | --- | --- | --- | --- | --- | --- | --- | --- | --- |
| **BPF** | case | f | 4 | 2.33 | 0.30 | 2.30 | 2.0 | 2.15 | 2.47 | 2.7 |
|  | case | m | 2 | 2.45 | 0.35 | 2.45 | 2.2 | 2.33 | 2.58 | 2.7 |
|  | control | f | 4 | 2.08 | 0.50 | 1.90 | 1.7 | 1.78 | 2.20 | 2.8 |
|  | control | m | 2 | 3.15 | 0.35 | 3.15 | 2.9 | 3.02 | 3.27 | 3.4 |
| **BPA** | case | f | 4 | 1.28 | 0.62 | 1.00 | 0.9 | 0.98 | 1.30 | 2.2 |
|  | case | m | 2 | 1.10 | 0.14 | 1.10 | 1.0 | 1.05 | 1.15 | 1.2 |
|  | control | f | 4 | 1.30 | 0.67 | 1.00 | 0.9 | 0.98 | 1.32 | 2.3 |
|  | control | m | 2 | 3.65 | 2.90 | 3.65 | 1.6 | 2.62 | 4.68 | 5.7 |
| **ClBPA** | case | f | 4 | 2.40 | 0.12 | 2.40 | 2.3 | 2.30 | 2.50 | 2.5 |
|  | case | m | 2 | 2.55 | 0.07 | 2.55 | 2.5 | 2.52 | 2.58 | 2.6 |
|  | control | f | 4 | 2.40 | 0.14 | 2.35 | 2.3 | 2.30 | 2.45 | 2.6 |
|  | control | m | 2 | 3.05 | 0.78 | 3.05 | 2.5 | 2.77 | 3.33 | 3.6 |

**Table S5. Study population descriptives and raw compound concentrations (ng/g) for the hypothalamic and white matter samples excluding the outlier (control without a matching white matter sample).**

|  | **HYP:case** | **WM:case** | **HYP:control** | **WM:control** |
| --- | --- | --- | --- | --- |
| n | 12 | 6 | 10 | 6 |
| Age (yrs) | 74.5 (13.8) | 73.6 (15.23) | 71.7 (15.9) | 71 (16) |
| Height (cm) | 162 (7.1) | 163 (7.7) | 169 (12.4) | 167 (7.4) |
| Body weight (kg) | 86.2 (7.9) | 87.1 (7.7) | 64.1 (9.7) | 63.8 (7.2) |
| BMI (kg/m^2^) | 32.8 (2.2) | 32.8 (2.5) | 22.2 (1.6) | 22.7 (1.5) |
| Brain weight (g) | 1132 (130) | 1146 (154) | 1193 (123) | 1189 (131) |
| Storage time (yrs) | 9.75 (5.71) | 8.0 (4.98) | 5.5 (2.1) | 7.3 (3.9) |

|  | **Status** | **n** | **Mean** | **SD** | **Median** | **Min** | **p25** | **p75** | **Max** |
| --- | --- | --- | --- | --- | --- | --- | --- | --- | --- |
| **Hypothalamus** | | | | | | | | | |
| **BPF** |  | 22 | 2.73 | 1.59 | 2.20 | 1.7 | 2.1 | 2.72 | 9.2 |
| **BPA** |  | 22 | 1.98 | 1.40 | 1.20 | 0.9 | 1.0 | 2.27 | 5.5 |
| **ClBPA** |  | 22 | 2.87 | 1.49 | 2.35 | 2.2 | 2.3 | 2.50 | 8.7 |
|  |  |  |  |  |  |  |  |  |  |
| **BPF** | case | 12 | 2.21 | 0.35 | 2.15 | 1.7 | 2.05 | 2.35 | 2.8 |
|  | control | 10 | 3.35 | 2.23 | 2.35 | 1.9 | 2.20 | 3.85 | 9.2 |
| **BPA** | case | 12 | 1.67 | 1.27 | 1.15 | 0.9 | 1.00 | 1.88 | 5.4 |
|  | control | 10 | 2.35 | 1.53 | 1.95 | 0.9 | 1.12 | 3.30 | 5.5 |
| **ClBPA** | case | 12 | 2.36 | 0.11 | 2.30 | 2.2 | 2.30 | 2.40 | 2.6 |
|  | control | 10 | 3.49 | 2.09 | 2.50 | 2.2 | 2.30 | 3.98 | 8.7 |

**Figure S1.** Bisphenols concentrations in human hypothalamus and white-matter/lipid enriched brain regions (n=12 pairs)


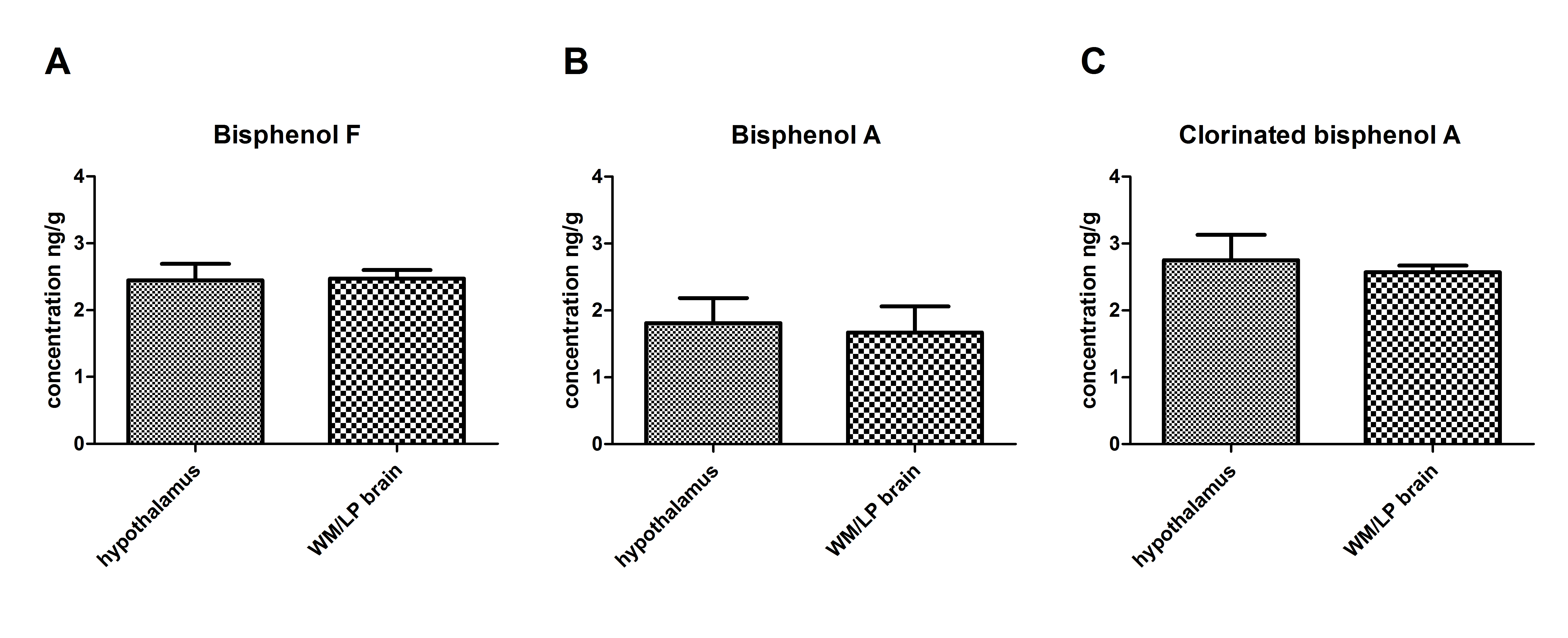


**Figure S2.** Bisphenols concentrations in human hypothalamus (HYP) and visceral and skin fat tissues (n=4).

**
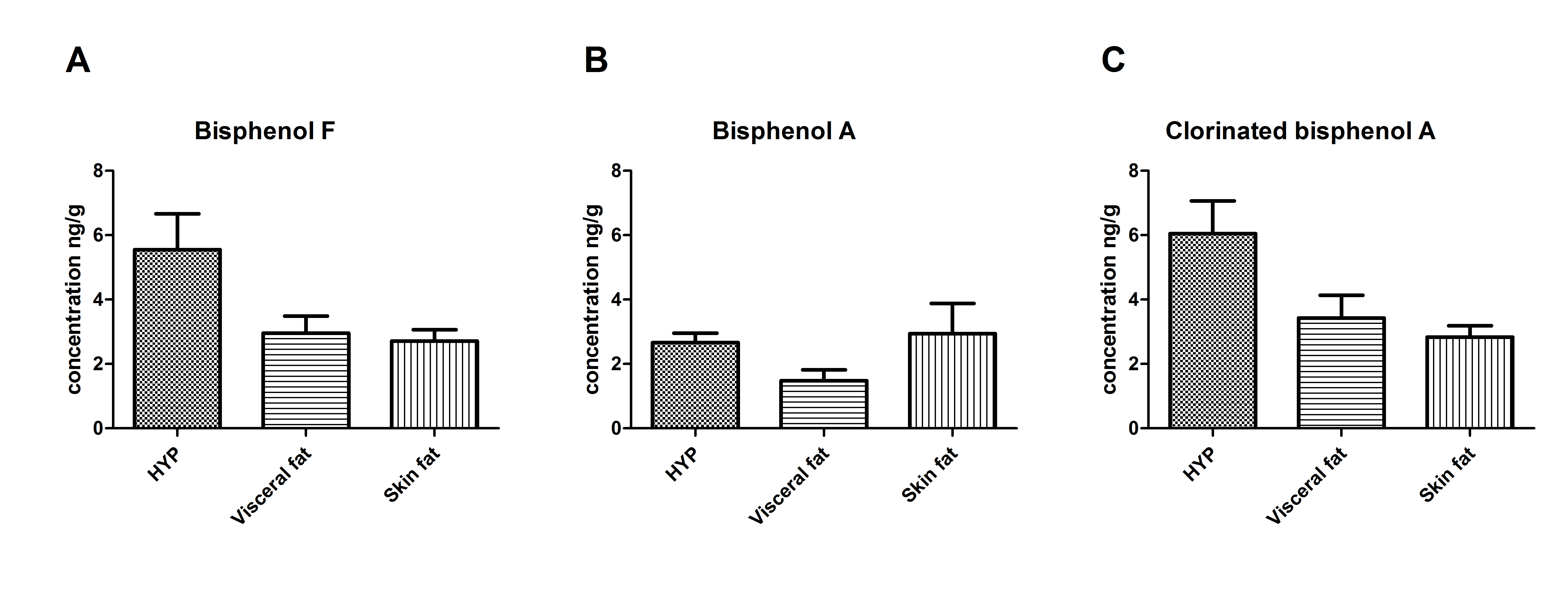
**
